# Infection With *Escherichia Coli* Pathotypes Is Associated With Biomarkers of Gut Enteropathy and Nutritional Status Among Malnourished Children in Bangladesh

**DOI:** 10.3389/fcimb.2022.901324

**Published:** 2022-07-06

**Authors:** Md. Amran Gazi, Md. Ashraful Alam, Shah Mohammad Fahim, Barbie Zaman Wahid, Shaila Sharmeen Khan, Md. Ohedul Islam, Md. Mehedi Hasan, S. M. Tafsir Hasan, Subhasish Das, Mustafa Mahfuz, Rashidul Haque, Tahmeed Ahmed

**Affiliations:** ^1^Nutrition and Clinical Services Division, International Centre for Diarrheal Disease Research, Bangladesh (icddr,b), Dhaka, Bangladesh; ^2^Infectious Diseases Division, International Centre for Diarrheal Disease Research, Bangladesh (icddr,b), Dhaka, Bangladesh; ^3^Liggins Institute, University of Auckland, Auckland, New Zealand; ^4^Faculty of Medicine and Life Sciences, University of Tampere, Tampere, Finland; ^5^James P. Grant School of Public Health, BRAC University, Dhaka, Bangladesh; ^6^Department of Global Health, University of Washington, Seattle, WA, United States

**Keywords:** *Escherichia coli*, diarrhea, malnutrition, environmental enteric dysfunction, Bangladesh

## Abstract

*Escherichia coli* (*E. coli*) pathotypes are the most common cause of diarrhea, especially in developing countries. Environmental Enteric Dysfunction (EED) is presumed to be the result of infection with one or more pathotypes and can affect intestinal health and childhood growth. We sought to investigate the association of *E. coli* pathotypes infection with biomarkers of EED and nutritional status among slum-dwelling malnourished children in Bangladesh. This study comprised a total of 1050 stunted and at risk of stunting children. TaqMan Array Card assays were used to determine the presence of *E. coli* pathotypes in feces. Prevalence of infection with EAEC was highest (68.8%) in this cohort of children, followed by EPEC (55.9%), ETEC (44%), Shigella/EIEC (19.4%) and STEC (3.2%). The levels of myeloperoxidase and calprotectin were significantly higher in EAEC (P=0.02 and P=0.04), EPEC (P=0.02 and P=0.03) and Shigella/EIEC (P=0.05 and P=0.02) positive participants while, only calprotectin was significantly higher in ETEC (P=0.01) positive participants. Reg1B was significantly higher in participants with EAEC (P=0.004) while, neopterin levels were significantly lower in ETEC (P=0.003) and Shigella/EIEC (P=0.003) positive cases. A significant positive relationship was observed between EAEC and fecal levels of Reg1B (β = 0.28; 95% CI = 0.12, 0.43; p-value<0.001). Besides, ETEC was found to be positively and significantly associated with the levels of calprotectin (β = 0.14; 95 percent CI = 0.01, 0.26; p-value=0.037) and negatively with neopterin (β = -0.16; 95% CI = -0.30, -0.02; p-value=0.021). On the other hand, infection with EPEC was found to be negatively associated with length-for-age (β = -0.12; 95% CI = -0.22, -0.03; p-value=0.011) and weight-for-age (β = -0.11; 95% CI = -0.22, -0.01; p-value=0.037). The study findings suggest that infection with certain *E. coli* pathotypes (EAEC and ETEC) influences gut health and EPEC is associated with linear growth and underweight in Bangladeshi children.

## Introduction

EED (Environmental Enteric Dysfunction) is a subclinical intestinal condition frequently found in underdeveloped and developing countries (Tickell et al., 2019). Poor sanitation, as well as microbial and parasitic contamination of food and water, are specifically associated with the disorder, which has also been linked to impaired cognitive function, poor linear growth and a diminished reaction to oral vaccinations (Tickell et al., 2019). EED is characterized by gut inflammation, increased intestinal permeability, and impaired small intestine absorption capacity. Intestinal biopsy remains to be the gold standard for diagnosing EED; however, the method is invasive and not attainable to perform on children (Syed et al., 2016). As a result, panels of biomarkers such as Neopterin (NEO), Alpha-1-antitrypsin (AAT), Myeloperoxidase (MPO), Calprotectin, and Reg1B have been proposed to assess EED (Bartelt et al., 2019; Tickell et al., 2019). MPO, NEO and calprotectin are markers of intestinal inflammation, while AAT is a measure of enteric protein loss and gut permeability (Fahim et al., 2018). Reg1B also known as regenerating family member 1 beta is a newly recommended marker that indicates epithelial tissue damage and subsequent regeneration in the small intestine (Budge et al., 2019). Infectious agents such as intestinal bacteria and parasites, as well as environmental disturbances, are responsible for EED (Salazar-Lindo et al., 2004). Intestinal infections are known to affect small intestine absorption and barrier function, subsequently contributing to the development of malnutrition, particularly in children (Rodríguez et al., 2011).

In resource- constrained settings, child malnutrition continues to be a significant risk factor for mortality and delayed long-term development (Ijarotimi, 2013). Early enteropathogen exposure has been linked to stunting in children; however, the role of specific enteropathogens has yet to be thoroughly investigated for Bangladeshi children. Previous studies have investigated a limited number of enteropathogens and their association with malnutrition in children from Bangladesh (Platts-Mills et al., 2017; Tickell et al., 2017; George et al., 2018; Lima et al., 2018). In underdeveloped nations, diarrhoeagenic strains of *Escherichia coli (E. coli)* are the prevalent cause of childhood diarrhea, with a higher frequency during the first two years of life (Gomes et al., 2016). They are subdivided into several pathotypes on the basis of their virulence genes which include enteroaggregative (EAEC), enterotoxigenic (ETEC), enteropathogenic (EPEC), shiga toxin–producing (STEC), and enteroinvasive (EIEC), among which EAEC, ETEC, and EPEC are mostly prevalent in developing countries (Gomes et al., 2016; Palit et al., 2021). Recent studies found the interaction effect of EAEC with other pathogens on gut inflammation and significant development impairments in children, with MPO concentrations being significantly higher with EAEC coinfection (Fahim et al., 2018; Lima et al., 2018). Another study showed that a subset of enteropathogens including EAEC, ETEC, and EIEC were associated with malnourished children aged <2 years in Bangladesh (Platts-Mills et al., 2017). Furthermore, a previous so found that the presence of ETEC in the stool of Bangladeshi pediatric population was associated with fecal concentration of calprotectin (George et al., 2018). Although there have been past studies that investigated potential relationships among particular pathotypes of *E. coli* and EED biomarkers, no studies included all pathotypes at once, to the best of our knowledge. Furthermore, considering the high prevalence of both *E. coli* infection and EED in this patient population, studies investigating how each illness interacts and affects patient outcomes is critical. Therefore, the aim of our study is to analyze the association between infections with *E. coli* pathotypes and biomarkers of EED and nutritional status (stunting, underweight and wasting) among malnourished children in Bangladesh.

## Materials and Methods

### Study Location, Study Design, and Ethics Statement

For this analysis, data was used from the Bangladesh Environmental Enteric Dysfunction (BEED) study, which was conducted in a resource constrained setting in Dhaka, Bangladesh. The BEED study was a nutrition intervention study targeting a local community where children either stunted [length-for age z score (LAZ) <2] or at risk of stunting [LAZ = −1 to −2] aged between 12 to 18 months were enrolled. The goals of the BEED study encompass determining the efficacy of a nutrition intervention in order to improve participants’ nutritional status, investigating the significance of enteric pathogens in the pathophysiology of EED and malnutrition, as well as the formation of a histological scoring method for EED detection and validating the score against non-invasive EED biomarkers. Severe anemia, severe acute malnutrition, presence of any hereditary disorder or malformation, tuberculosis, diarrhoea, presence of any severe or chronic disease and another family member already enrolled in the BEED study were all exclusion criteria for enrollment in the BEED study. The BEED study’s methodology was previously published (Mahfuz et al., 2017). The research protocol (protocol no.: PR-16007) was reviewed and accepted by the Institutional Review Board of the International Center for Diarrheal Disease Research, Bangladesh (icddr,b). Written informed consent was taken from all parents or guardians of the children before being enrolled in the study.

### Data and Sample Collection

At the time of enrollment, field workers obtained socio-demographic and household information from the participants’ parents or caregivers. The trained field workers used standard operating methods to measure anthropometry. We provided field employees with refresher training and evaluated intra-class correlation coefficient (ICC) every three months to verify the consistency of anthropometric measurements from one rater to the next. With a coefficient greater than 0.9 for each of the scales, such training leads to a significant improvement in raters’ anthropometric measures at the field location. The WHO anthropometry software was used to generate nutritional status indicators such as length-for-age z (LAZ), weight-for-age z (WAZ), and weight-for-length z (WLZ) scores. For laboratory assays, non-diarrheal stool and blood samples were also taken at the baseline. Samples of feces were collected without the use of any fixatives. To extract the plasma from blood samples, centrifuges were used for 10 minutes at 4000 rotations per minute. Prior to analysis, all aliquots of plasma and stools were frozen at -80°C.

### Laboratory Analysis

Laboratory experiments were performed at icddr,b. Commercial ELISA kits were used to assess plasma biomarkers such as alpha-1-acid glycoprotein (AGP) (Alpco, Salem, NH), C-reactive protein (CRP) (Immundiagnostik, Bensheim, Germany), and ferritin (ORGENTEC Diagnostika GmbH, 55129 Mainz, Germany). The quantity of zinc in the plasma was determined using atomic absorption spectrometry. MPO (Alpco, Salem, New Hampshire), AAT (Biovendo Chandler, North Carolina), NEO (GenWay Biotech, San Diego, California), Calprotectin (BUHLMANN fCAL, Schonenbuch, Switzerland) and Reg1B (TechLab, Blacksburg, Virginia) were among the EED fecal biomarkers studied by ELISA. A quantitative PCR test employing TaqMan Array Cards (TAC) by Applied Biosystems (Life Technologies Corporation, Carlsbad, CA) was used to detect the presence of *E. coli* pathotypes and other enteropathogens in stool samples. It is a 384-well singleplex probe-based real-time PCR test for identifying pathogens in stool samples. Firstly, nucleic acid was extracted using a modified process that includes bead beating with the QIAamp Fast DNA Stool Mini Kit (Qiagen, Hilden, Germany) in this investigation (Ijarotimi, 2013). During nucleic acid extraction, two external controls, phocine herpes virus and MS2 bacteriophage, were added to samples to evaluate the efficacy of extraction and amplification. Contamination was monitored using extraction blanks and no-template controls. The established cut-off for detecting pathogens was 35 Ct-value (cycle of threshold) in TAC assay. As a result, a Ct value of 35 was assumed to indicate pathogen positivity. The performance and inter laboratory reproducibility of the TAC approach have been previously described (Liu et al., 2013; Platts-Mills et al., 2017).

### Variables Used in This Analysis

The fundamental goal of this study was to demonstrate if the presence of *E. coli* pathotypes in the feces were significantly associated with increased levels of EED biomarkers and nutritional status in young children. Hence, *E. coli* pathotypes were chosen to be the exposure variable. This binary variable was categorized based on the presence of specific *E. coli* pathotypes derived from the stool TAC findings. The outcome variables in the current study included fecal biomarkers (e.g. NEO, MPO, Calprotectin, AAT and Reg1B) and nutritional status indicators (e.g. LAZ, WAZ, and WLZ). Age, sex, wash practices, WAMI index (water/sanitation, assets, maternal education, and income), maternal height, inflammatory biomarkers (AGP, CRP), zinc and ferritin were all covariates for EED model while age, sex, WAMI Index, wash indices, EED biomarkers (AAT, MPO, NEO, calprotectin, Reg1B), maternal height, zinc, and ferritin were considered as covariates for nutritional status modeling in this study.

### Statistical Analyses

The demographic and socioeconomic variables were represented using median with inter-quartile ranges (IQR) for asymmetric continuous data, and mean with standard deviation for symmetric continuous variables. Frequency measures were used for categorical variables. To assess the differences of EED biomarkers between *E. coli* pathotypes positive and negative groups, t-test or Mann-Whitney U test was performed.

All the fecal biomarker concentrations (Reg1B, AAT, MPO, NEO and Calprotectin) were log transformed and normalized before being included in the model. Using multivariable linear regression, we analyzed the relationship between *E. coli* pathotypes and EED biomarker concentrations, as well as the relationship between *E. coli* pathotypes and nutritional status indicators (LAZ, WAZ, and WLZ) of the children. On the basis of existing literature, we first created a conceptual framework and chose the covariates accordingly while all the individual models were adjusted by those covariates. R 3.5.3 was used to conduct the statistical analysis. A probability of 0.05 was deemed as statistically significant.

## Results

**Figure 1:**
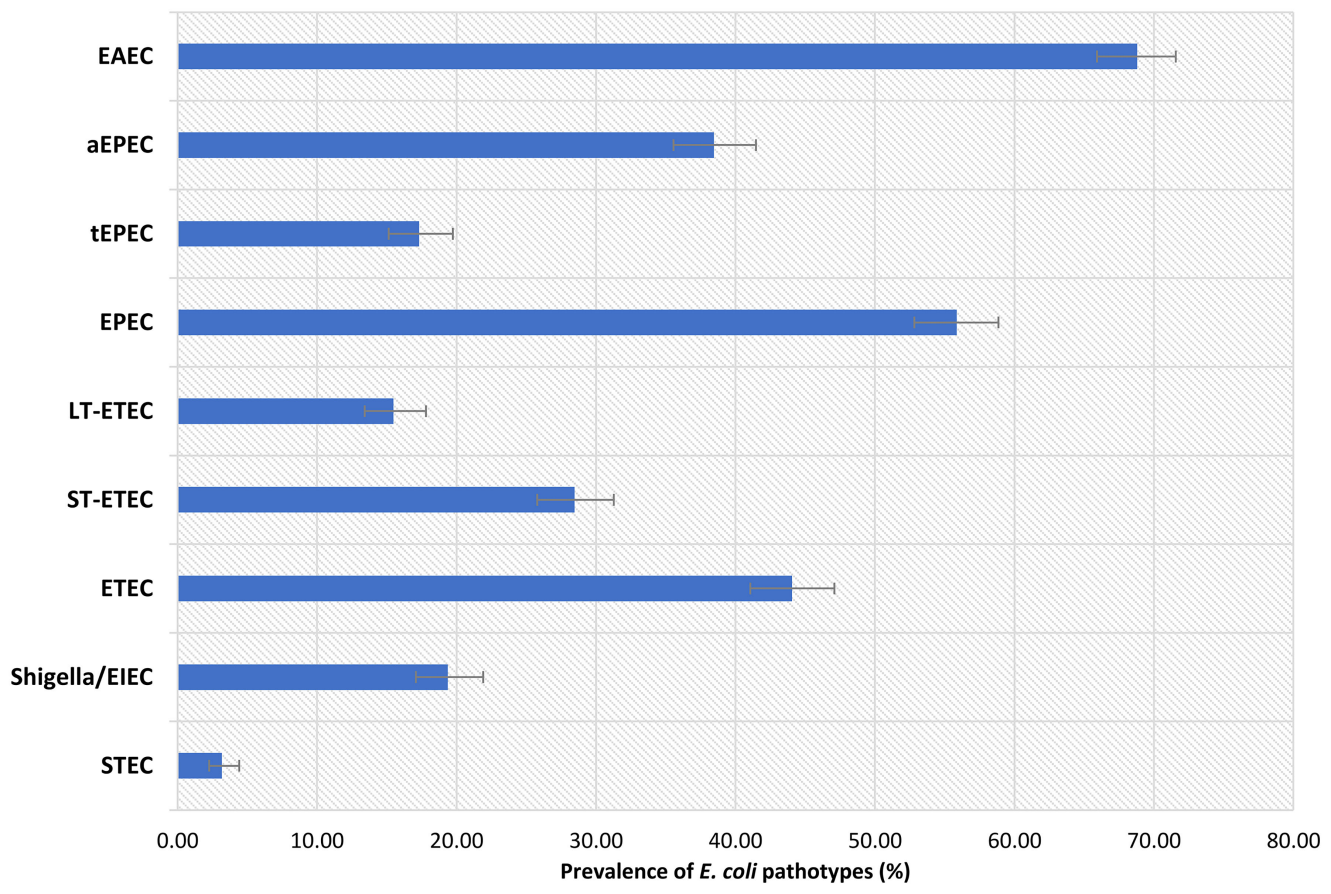
Prevalence of *E. coli* pathotypes among the participants.

In this study, a total of 1050 children were included. Of these children, 525 were stunted, while another 525 were on the verge of being stunted. The male to female ratio was about equal, and the children’s mean ( ± SD) age was 14.8 (2.1) months. The baseline characteristics of the enrolled children are listed in **Table 1**. Prevalence of EAEC was highest among the participants (68.8%), following EPEC, ETEC, Shigella/EIEC and STEC. Prevalence of heat-stable (ST)-ETEC was higher (28.5%) than heat-labile (LT)-ETEC (15.5%). On the other hand, 38.5% participants were found positive for atypical EPEC (aEPEC) and 17.3% for typical EPEC (tEPEC). The prevalence of *E. coli* pathotypes is presented in **Figure 1**.

**Table 1:** Baseline characteristics of children at enrollment (n = 1050).

Indicators	Values
Child age in months, mean (SD)	14.8 (2.14)
Female sex, n (%)	527 (50.2%)
Child weight in Kg, mean (SD)	8.23 (0.915)
Child height in cm, mean (SD)	72.4 (2.93)
Weight for age z-score, mean (SD)	-1.66 (0.845)
Length for age z-score, mean (SD)	-2.15 (0.774)
Weight for length z-score, mean (SD)	-0.834 (0.890)
Treated water, n (%)	569 (54.3%)
Always wash hand with soap after child defecation, n (%)	686 (65.5%)
Always wash hand with soap before preparing food, n (%)	122 (11.6%)
Always wash hand with soap after using toilet, n (%)	779 (74.3%)
Always use toilet paper, n (%)	206 (19.7%)
Improved sanitation, n (%)	637 (60.8%)
Monthly family income in USD, median (IQR)	167 [121, 239]
Asset score, median (IQR)	4 [2, 5]
Maternal education in years, median (IQR)	5 [2, 8]

IQR, Inter-quartile range; SD, standard deviation.

### Distribution of Fecal Biomarkers Among Children Infected With *E. Coli* Pathotypes

There was no difference in the distribution of AAT and NEO levels among the children with and without EAEC infection. However, we observed a significantly higher fecal concentration of MPO (p=0.02), calprotectin (p=0.04) and Reg1B (p=0.004) in children tested positive for EAEC compared to those who were uninfected. The median (interquartile range [IQR]) levels of MPO, calprotectin and Reg1B were 2340 (1170, 5460) ng/mL, 522 (289, 911) μg/g and 72.3 (41.1, 98.8) μg/mL, respectively for EAEC positive participants. Similarly, the median (IQR) concentration of MPO and calprotectin were 2410 (1260, 5050) ng/mL and 529 (307, 880) μg/g, respectively in EPEC positive participants which was significantly higher compared EPEC negative participants (p-value =0.02 and p-value =0.03, respectively). Higher median (IQR) concentration of MPO 2490 (1170, 6400) ng/mL and calprotectin 584 (338, 886) μg/g was also found for Shigella/EIEC positive children (p-value =0.05 and p-value =0.02, respectively) while median (IQR) concentration of NEO 1250 (719, 2610) was significantly lower in these participants (p-value =0.003). Likewise, the median (IQR) calprotectin 555 (298, 920) was significantly higher and NEO 1490 (755, 2680) nmol/L was significantly lower in the stool samples of ETEC positive malnourished children (p-value =0.01 and p-value =0.003, respectively). Distribution of fecal biomarkers according to infection with *E. coli* pathotypes are presented in **Table S1** and **Figures S1– S5** (**Supplementary Figure File**).

### Coinfection Among *E. Coli* Pathotypes and Other Enteropathogens

Coinfection among *E. coli* pathotypes and other enteric pathogens were detected in our study population. For instance, EAEC is significantly coinfected with ETEC (p=0.008), EPEC (p=<0.001) and *Cryptosporidium* (p=0.004) while EPEC is additionally associated with STEC (p=<0.001). ETEC and *Shigella*/EIEC is significantly codetected with *Campylobacter* (p=0.001) while STEC is significantly coinfected with *Giardia* (p=0.045) and *Bacteroides fragilis* (p=0.026). Coinfection of *E. coli* pathotypes and other pathogens data were presented in **Table S2**.

### Association of Infection With *E. Coli* Pathotypes and Fecal Biomarkers of EED

**Figure 2:**
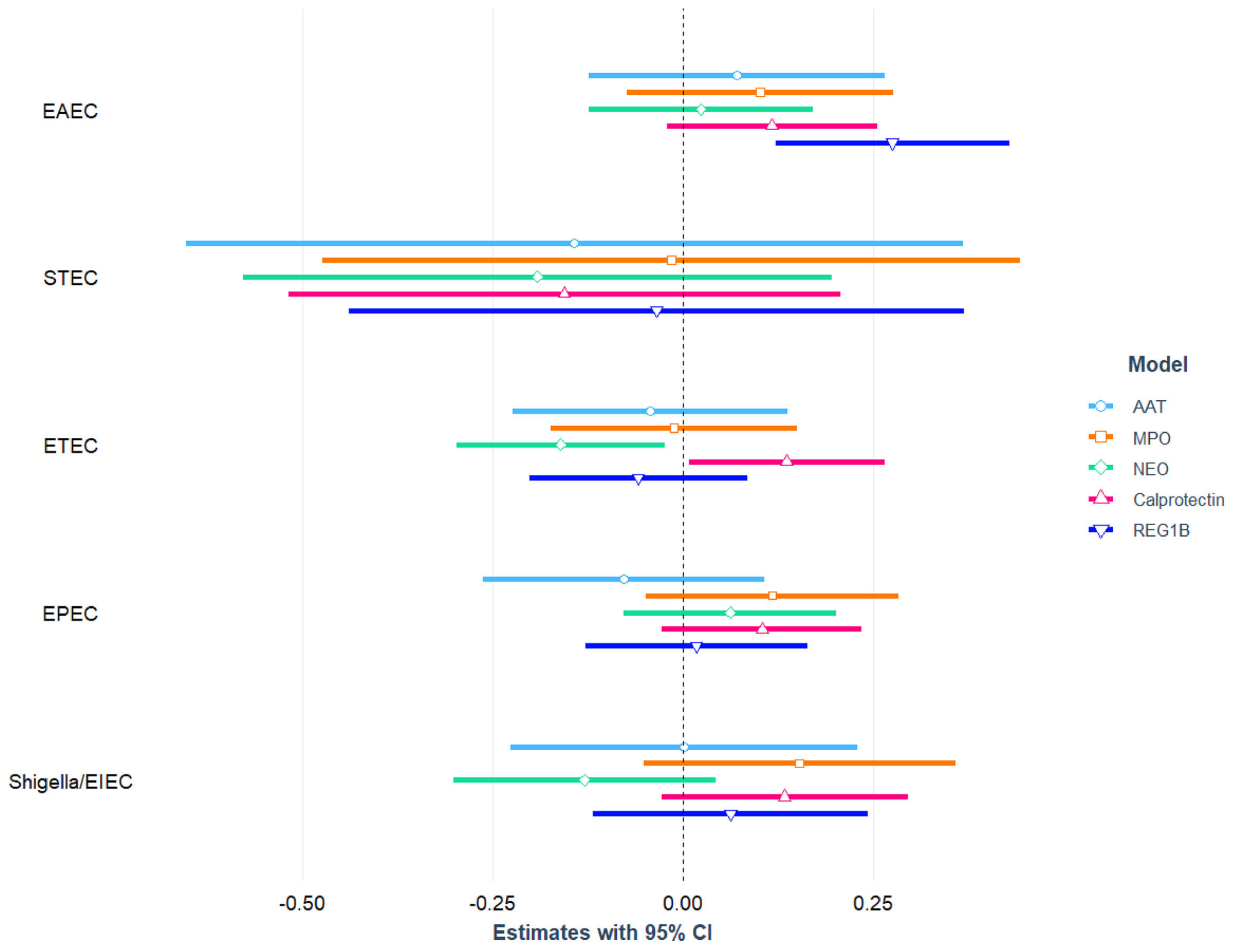
Association between *E. coli* pathotypes and fecal biomarkers of EED in children.

The association between *E. coli* pathotypes and fecal biomarkers of EED is depicted in **Figure 2** and also presented in **Table S3**, **S4** with covariates. After correcting for potential confounders, in multivariable linear regression analysis, EAEC infection was positively linked with fecal Reg1B concentrations (β = 0.28; 95 percent CI = 0.12, 0.43; p-value<0.001), and the association was statistically different. Similarly, in an adjusted model, a statistically significant positive association was found between ETEC infection and fecal calprotectin concentrations (β = 0.14; 95 percent CI = 0.01, 0.26; p-value=0.037), while an inverse relationship was found with NEO (β= -0.16; 95 percent CI = -0.30, -0.02; p-value=0.021). We further parsed ETEC into ST-ETEC and LT-ETEC and found LT-ETEC to be significantly and positively associated with calprotectin while negatively associated with NEO. None of the other *E. coli* pathotypes (EPEC, Shigella/EIEC, or STEC) were found to be linked to any of the EED biomarkers.

### Association of Infection With *E. Coli* Pathotypes and Nutritional Status of the Children

**Figure 3:**
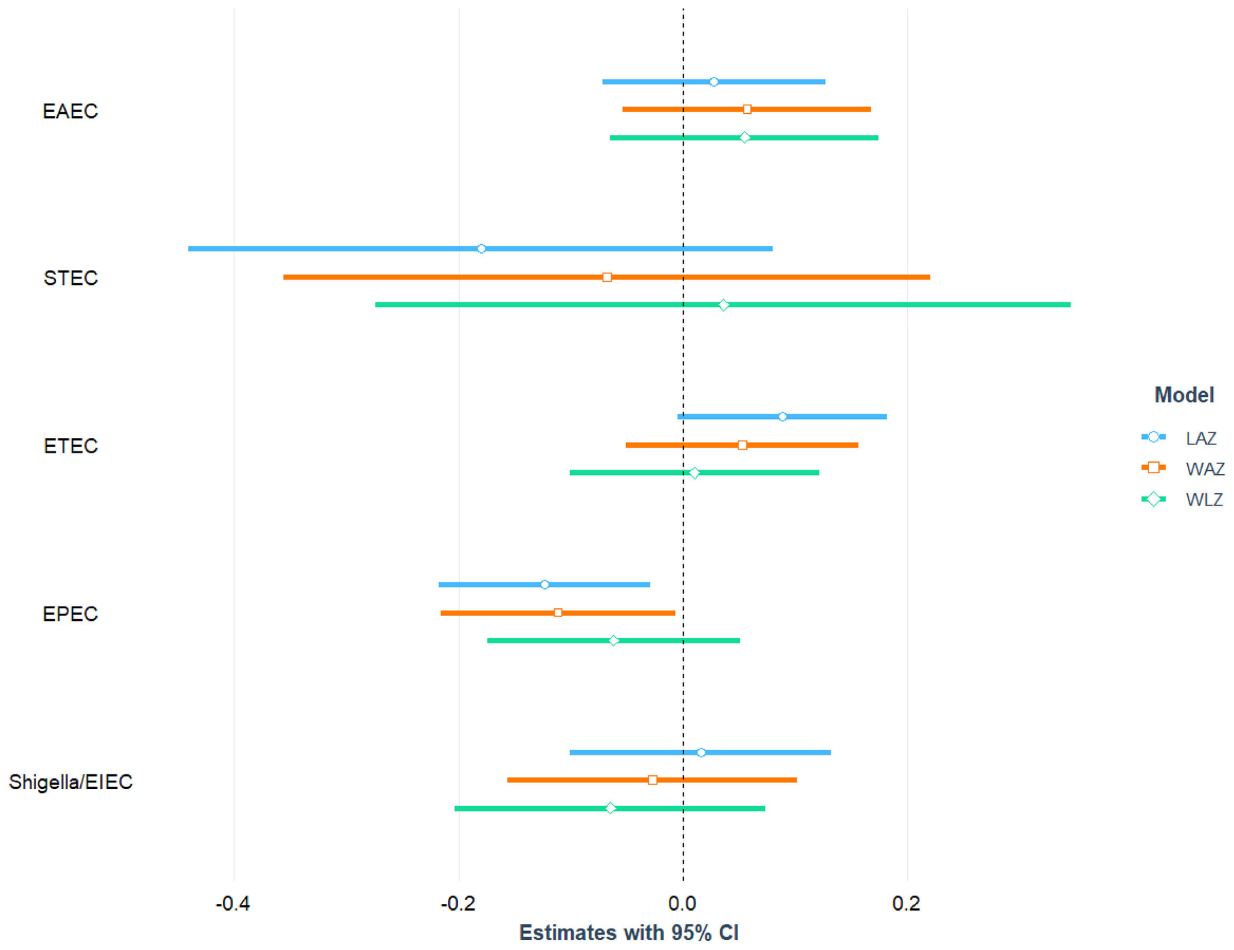
Association between *E. coli* pathotypes and nutritional status of children.

After adjusting for age, sex, mother’s education, maternal height, crowded living conditions, educational status of household head, water treatment, separate space for kitchen, mothers washing hands after toilet, and feces, infection with EPEC was found to be significantly and negatively associated with LAZ (= -0.12; 95% CI = -0.22, -0.03; p-value=0.011) and WAZ (β = -0.11; 95% CI = -0.22, -0.01; p-value=0.037). After adjusting for the above-mentioned confounding variables, no statistically significant relationship was detected between EPEC infection and the children’s WLZ score. We categorized EPEC into aEPEC and tEPEC and revealed that aEPEC is both significantly and negatively associated with LAZ score. Furthermore, a significant positive association was found between ST-ETEC and LAZ score. No other *E. coli* pathotypes (EAEC, ETEC, Shigella/EIEC and STEC) were associated with LAZ, WAZ and WLZ score of the study children. The association of *E. coli* pathotypes with nutritional status of the children is depicted in **Figure 3** and also presented in **Tables S5**, **S6** with covariates.

## Discussion

Undernutrition is still a major issue around the world, with diarrhea and environmental enteropathy being implicated as contributing factors. In addition, a rate-limiting step in achieving normal nutrition may be diminished absorptive function due to recurrent enteric infections (Guerrant et al., 2008). In these circumstances, intestinal pathogenic *E. coli* plays an important role as one of the large causative groups of these infections worldwide (Rojas-Lopez et al., 2018). Most prior studies in similar settings primarily investigated and revealed an association between a limited number of *E. coli* pathotypes and malnutrition. In this current study, the five prevalent pathotypes of *E. coli* were investigated as to their association with EED biomarkers and nutritional status among malnourished children in Bangladesh. Our study depicted that in Bangladeshi children, infection with particular *E. coli* pathotypes (EAEC and ETEC) affects gut health biomarkers while, EPEC is negatively linked to linear growth deficits and underweight in children living in a slum of Bangladesh. Our results also showed that infection with EAEC was most prevalent (approx. 68%), followed by EPEC (55.9%), ETEC (44%), Shigella/EIEC (19.4%) and STEC (3.2%) respectively among our study children.

Although *E. coli* constitutes an important unit of normal gut bacterial flora, several studies have shown the strains to harbor distinct virulence factors and to be diarrheagenic (Nataro and Kaper, 1998; Johura et al., 2017). Previous studies showed that EAEC, ETEC and EPEC seem to be major causes of infantile diarrhea in the developing world (Abba et al., 2009; Clements et al., 2012; Jafari et al., 2012). A recent study conducted among Bangladeshi children dwelling in an urban slum found that the most frequently found pathogens in the children with EED and healthy children were EAEC, ETEC, and EPEC (Chen et al., 2020). Prior study in Bangladesh also showed that the detection of EAEC was highest in both severely acute malnourished and well-nourished groups followed by EPEC and ETEC in the malnourished group which is in line with our current findings (Palit et al., 2021). Moreover, a systematic review of the ailment in underdeveloped countries depicted that EAEC, ETEC, and EPEC are the primary pathogens and are accountable for 30-40% of all persistent diarrhea among children (Abba et al., 2009). Furthermore, the current analysis exhibits higher prevalence of aEPEC compared to tEPEC. For many years, tEPEC was considered to be a primary cause of infantile diarrhea in developing countries, but it was considered rather unusual in industrialized countries. Yet, recent evidence suggests that, even in instances of persistent diarrhea, aEPEC is more prevalent than tEPEC in both developed and developing nations (Ochoa et al., 2008). Besides, ETEC expressing the heat-stable enterotoxin was predominant in this study population. Previous Studies conducted in Bangladesh reported higher prevalence of ST-ETEC and lower LT-ETEC among diarrheal patients (Qadri et al., 2000; Qadri et al., 2007).

The greater prevalence of E. coli pathotypes found in our research may be related to coinfection among *E. coli* pathotypes or with other enteric pathogens. For instance, EAEC and STEC were found to be significantly associated with *Cryptosporidium*, and *Bacteroides fragilis*, respectively, while *Shigella*/EIEC and STEC were significantly associated with *Campylobacter*. Similarly, *Giardia* was significantly coinfected with STEC. There were also coinfections amongst *E. coli* pathotypes. In various diarrhoeal studies around the world, dual infections with enteric pathogens have been increasingly recognized (Zhang et al., 2016; Ledwaba et al., 2018; Pijnacker et al., 2019). Recent studies of diarrheal etiology have also begun to describe coinfections, suggesting patients infected with these pathogens may experience more severe disease outcomes than single infections (Bhavnani et al., 2012). Pathogens are continually developing, and interactions between microbes in the host might result in pathogens competing for the same niche, resulting in increasing virulence. Previous research demonstrated that, ETEC promotes EPEC coinfection with elevated inflammatory responses *in vitro* and also *in vivo* with C57Bl/6 mice developing severe diarrhea (Ledwaba et al., 2020). On the other hand, EAEC and EPEC coinfections have antagonistic effects with reduced inflammatory responses *in vitro* (Ledwaba et al., 2020).The study revealed a significant positive association between fecal concentration of Reg1B and EAEC. Reg1B was also found to be significantly higher in EAEC infected participants than the children who were negative for EAEC. Prior evidence revealed association of Reg1B with enteric infections in Bangladeshi children (Fahim et al., 2021). Reg1B proteins play a crucial role in cell differentiation and proliferation in the gastrointestinal system (Peterson et al., 2013). Inflammation of the intestine and conditions such as active inflammatory bowel disease, pseudomembranous colitis, and amebiasis cause Reg1B to increase (Granlund et al., 2011; Peterson et al., 2011; van Beelen Granlund et al., 2013). Because Reg1B has been linked to cell regeneration and intestinal inflammation, it is possible that an earlier inflammation produced by EAEC activated Reg1B’s action. According to a study constituting children under the age of five from Bangladesh and Peru, increased levels of Reg1B were associated with stunting (Peterson et al., 2013). In the multivariable model, our results also suggested a positive association of ETEC and LT-ETEC with calprotectin. This is consistent with previous literature demonstrating that a pediatric population with ETEC infection had a significantly higher fecal calprotectin concentration (George et al., 2018). The study also showed a significant positive association between the presence of other enteric pathogens in stool and elevated fecal calprotectin concentrations (George et al., 2018). Different studies showed that LT-ETEC was associated with increased MPO and malnutrition (Platts-Mills et al., 2017; Iqbal et al., 2019). Another study comprising infants and children between three months to four years of age in Southern India demonstrated that fecal calprotectin concentrations were higher in samples with multiple enteropathogens (Praharaj et al., 2018). The current analysis additionally observed that calprotectin levels were significantly higher when the participants were infected with EAEC, EPEC and ETEC compared to the negative participants. Several pathological conditions can cause infection or inflammation of the gut mucosa which leads to an increase in permeability of the mucosa. This leads to enhanced migration of monocytes and granulocytes towards chemotactic substances in the bowel (De Oliveira et al., 2016). Besides, bacterial components obtained from the intestinal lumen act as stimuli for the release of mediators for instance calprotectin from granulocytes and monocytes, and therefore the amount of calprotectin increases in the feces (Bjarnason, 2017). Thus, the presence of calprotectin in feces is a consequence of an infection or an inflammatory process and migration of neutrophils into the gastrointestinal tissue (Bjarnason, 2017). Furthermore, a significant negative association was found between ETEC or LT-ETEC and the levels of NEO. Besides, NEO levels were significantly lower in ETEC and Shigella/EIEC positive participants than their counterparts. Although it seems to be counter-intuitive to the commonly found association of infection with enteropathogens and changes in inflammatory biomarkers, a previous study showed that EAEC pathogen codetection was linked with lower fecal NEO levels (Lima et al., 2018). Lower levels of NEO were also found to be associated with *Giardia* and *Yersinia enterocolitica* (Kosek et al., 2017). In addition, codetection of one or more *E. coli* pathotypes and other pathogens was found in our investigation, which could elucidate why ETEC and Shigella/EIEC positive participants exhibited lower NEO levels. According to findings from a prior mouse model study, coinfections can have either synergistic or antagonistic effects (Ledwaba, 2020). However, further research is required to establish the firm link how infection with individual *E. coli* pathogenic strain cause the changes in NEO. Moreover, EAEC and EPEC positive participants has shown a significant increase in MPO levels. However, MPO was not significantly associated with any of the *E. coli* pathotypes in the multivariable model. An earlier study confirms a higher frequency of enteric pathogens in stool was linked to higher levels of fecal MPO and this biomarker has been employed as an enteropathy biomarker (George et al., 2018). In an earlier murine model, MPO was increased during the acute phase of EPEC infection, which is in line with our current finding (Ledwaba et al., 2020). Findings from MAL-ED birth cohort studies also revealed positive association of EAEC with MPO levels (Kosek et al., 2017; Lima et al., 2018).

The results also revealed a significant negative association between EPEC with linear growth (as measured by LAZ) and underweight (as measured by WAZ) and aEPEC with LAZ among Bangladeshi children. EPEC is one of the most common enteropathogenic microorganisms found in the stools of infantile diarrhea. However, several studies have found EPEC with similar frequency among diarrheal and non- diarrheal samples (Ochoa and Contreras, 2011). EPEC strains are able to stick to human intestinal tissue where they induce a typical lesion that leads to the dissolution of the brush border membrane and loss of microvilli structures. The presence of this damage is related to fluid secretion and imbalance of the digestive-absorptive enzymatic system, which is responsible for the persistent diarrhoea associated with malabsorption of nutrients (Neto and Scaletsky, 2000). EPEC infection has also been shown to damage the ileal and colonic mucosa’s tight junction barrier function and increase intestinal permeability (Ledwaba et al., 2020). These conditions in the gut can lead to food intolerance and contributes to long term conditions such as malnutrition (Ledwaba et al., 2020). EPEC infection is often associated with malnutrition of the host, especially in individuals experiencing severe and prolonged infection, and/or in those who are nutritionally compromised. Its association with malnutrition was first described in Brazilian children (Neto and Scaletsky, 2000). In a previous murine EPEC infection model, in an acute symptomatic phase of the disease, growth impairment and diarrhea were demonstrated in EPEC infected mice, and this was accompanied by intestinal damage and inflammation (Neto and Scaletsky, 2000). In addition, EPEC infection of human intestinal epithelial cells leads to inhibition in vitamin C and thiamin (vitamin B1) uptake that may contribute to micronutrient deficiency and subsequent malnutrition in children (Ashokkumar et al., 2009; Heskett et al., 2020). Apart from EPEC, other enteric infections were considered to be significantly linked with EED and impaired growth in rural Bangladesh in an earlier study (George et al., 2018). An observational study showed that enteric infections contributed to the nutritional decline during the first year of life among children in low- income settings (Mondal et al., 2012). A previous case-control study conducted among Bangladeshi pediatric population depicted an association between infection with a number of enteric pathogens (*Campylobacter*, EAEC, LT-ETEC, *Shigella*/EIEC, norovirus genogroup II, and *Giardia*) and malnutrition (Platts-Mills et al., 2017). Overall, the results from our current research hence provide valuable insights into the complex pathogenesis of EPEC infection and child malnutrition in Bangladesh. However, future research should consider the inclusion of metabolomic biomarkers, host metabolism, microbiome and growth phenotypes data to expand the understanding of how *E. coli* pathotypes affect the nutritional status in these populations.

Our study has several limitations. First, the children in this study were all malnourished inhabited in low-resource setting. Therefore, a control group comprising healthy children from better socio-economic condition would be worthwhile to understand the impact of *E. coli* pathotypes on intestinal health biomarkers of children and their nutritional status. Second, we could not adjust some important variables in our analysis for example, breast feeding status, birth weight, pathogen burden, maternal height those have the impact on childhood growth according to the previous literatures.

In conclusion, our findings imply that EPEC infection is associated with underweight and linear growth impairment in Bangladeshi children. In addition, EAEC and ETEC is associated with gut health biomarkers, suggesting relevant biological pathways involved during these infections. The current research can help further explore mechanisms involved in EED and nutritional status with three prevalent *E. coli* pathotypes for instance, EPEC, EAEC and ETEC pathogenesis and perhaps facilitate the development of therapeutic interventions.

## Data Availability Statement

The original contributions presented in the study are included in the article/**Supplementary Material**. Further inquiries can be directed to the corresponding authors.

## Ethics Statement

The studies involving human participants were reviewed and approved by Research Review Committee (RRC) and Ethical Review Committee (ERC), International Centre for Diarrhoeal Disease Research, Bangladesh. Written informed consent to participate in this study was provided by the participants’ legal guardian/next of kin.

## Author Contributions

MG, MA and TA originated the idea for the study and led the protocol design. SK, MI, MH and RH performed and supervised the sample analysis. MG, MA, SF, BW, SMH, SD and MM wrote the manuscript and were involved in data analysis. MG, MA and TA interpreted the results. All authors read and approved the final manuscript. All authors contributed to the article and approved the submitted version.

## Funding

This study was funded by the Bill and Melinda Gates Foundation. The investment ID was OPP1136751. (https://www.gatesfoundation.org/How-We-Work/Quick-Links/GrantsDatabase/Grants/2015/11/OPP1136751). This research protocol is funded by the Bill & Melinda Gates Foundation.

## Author Disclaimer

The funders had no role in the study design; collection, analysis, and interpretation of data; preparation, review, or approval of the manuscript; and decision to submit the manuscript.

## Conflict of Interest

The authors declare that the research was conducted in the absence of any commercial or financial relationships that could be construed as a potential conflict of interest.

## Publisher’s Note

All claims expressed in this article are solely those of the authors and do not necessarily represent those of their affiliated organizations, or those of the publisher, the editors and the reviewers. Any product that may be evaluated in this article, or claim that may be made by its manufacturer, is not guaranteed or endorsed by the publisher.
