# Supplementary material for: Infection With Escherichia Coli Pathotypes Is Associated With Biomarkers of Gut Enteropathy and Nutritional Status Among Malnourished Children in Bangladesh
Source: Front Cell Infect Microbiol. 2022 Jul 6;12:901324. doi: 10.3389/fcimb.2022.901324 (PMC9299418; doi:10.3389/fcimb.2022.901324)
Supplement: Supplementary file 1 [file DataSheet_1.docx]

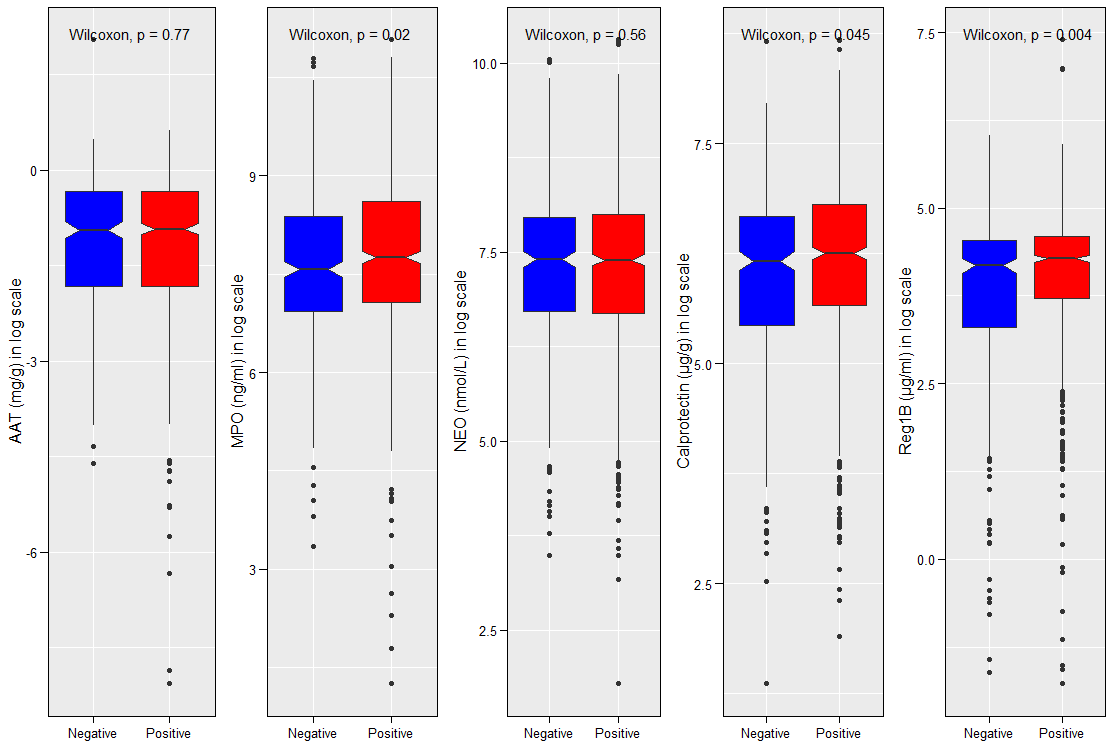


**Figure S1:** Distribution of fecal biomarkers in children (n = 1030) with and without EAEC infection. A1AT, alpha-1 antitrypsin; MPO, myeloperoxidase; NEO, neopterin; Reg1B, regenerating family member 1 beta; EAEC, enteroaggregative *E. coli*.


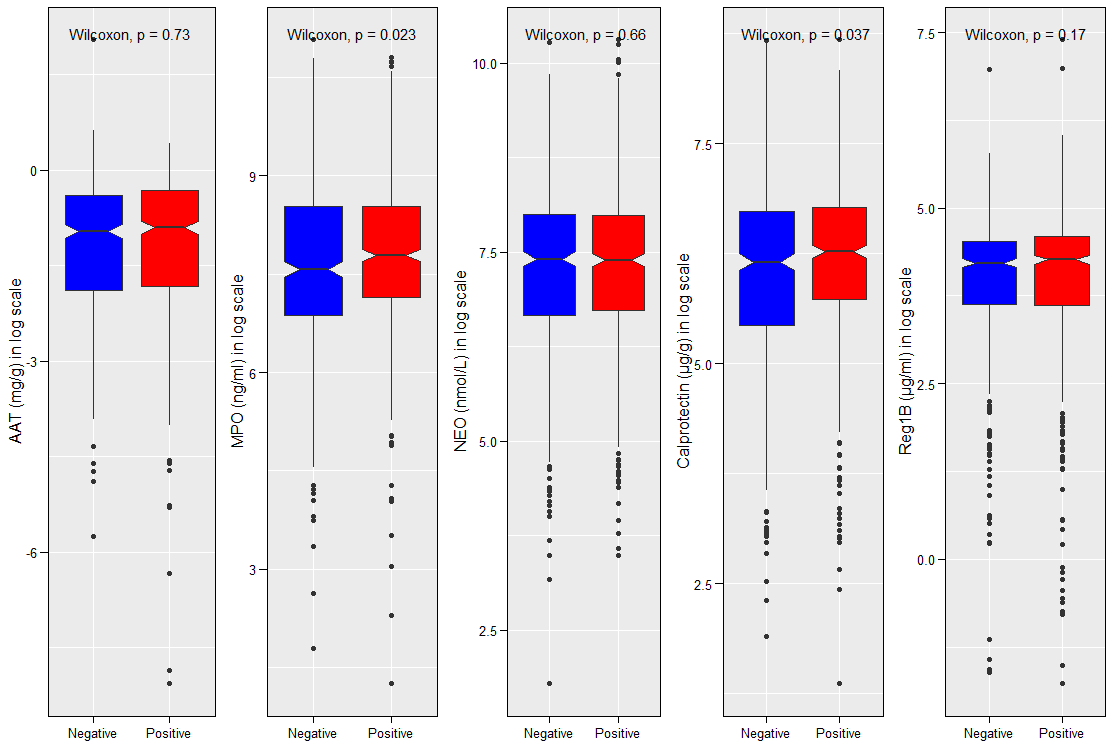


**Figure S2:** Distribution of fecal biomarkers in children (n = 1030) with and without EPEC infection. A1AT, alpha-1 antitrypsin; MPO, myeloperoxidase; NEO, neopterin; Reg1B, regenerating family member 1 beta; EPEC, enteropathogenic *E. coli*.


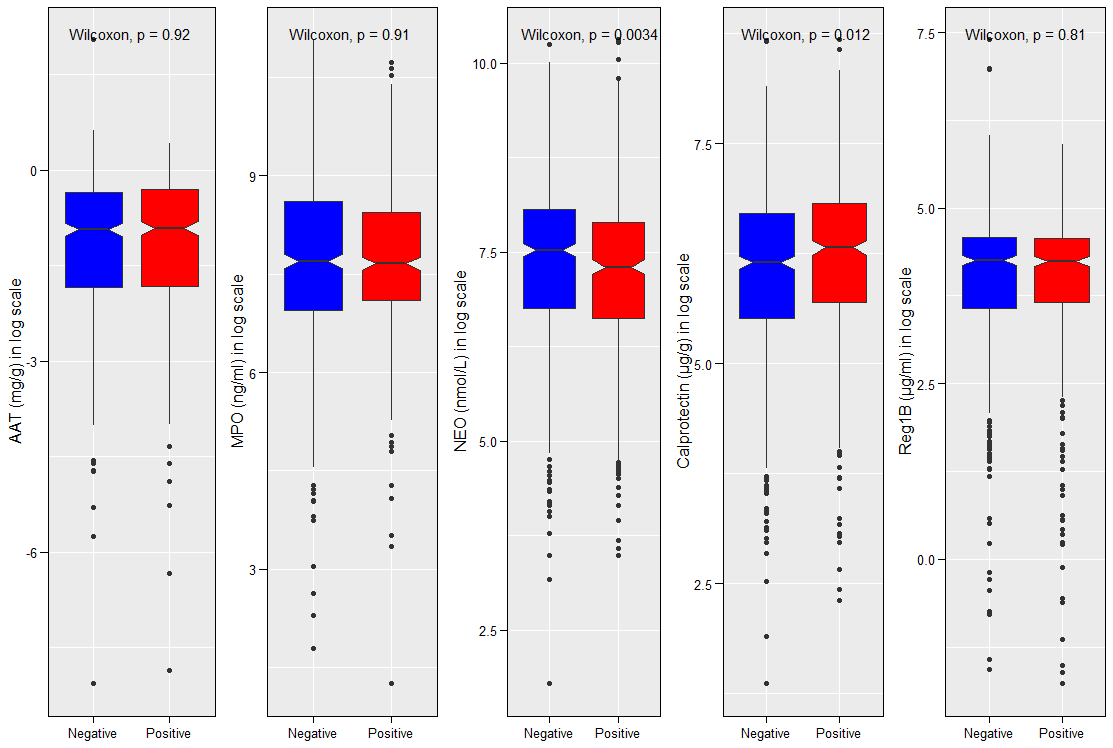


**Figure S3:** Distribution of fecal biomarkers in children (n = 1030) with and without ETEC infection. A1AT, alpha-1 antitrypsin; MPO, myeloperoxidase; NEO, neopterin; Reg1B, regenerating family member 1 beta; ETEC, enterotoxigenic *E. coli*.


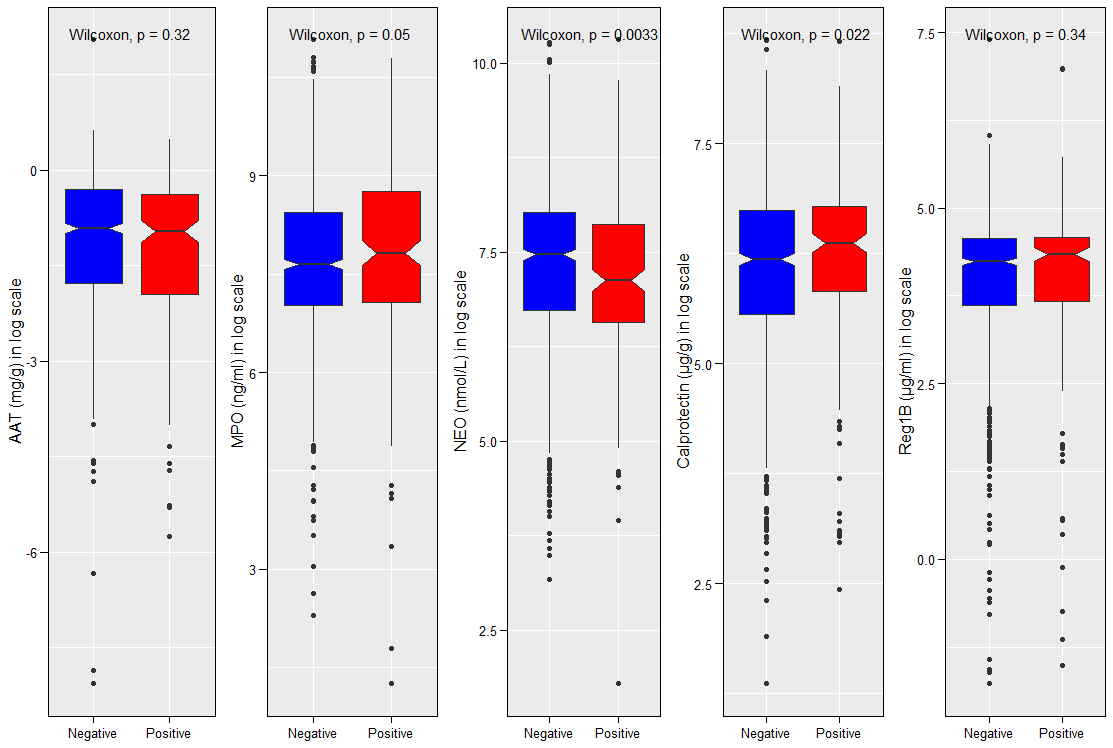


**Figure S4:** Distribution of fecal biomarkers in children (n = 1030) with and without Shigella/EIEC infection. A1AT, alpha-1 antitrypsin; MPO, myeloperoxidase; NEO, neopterin; Reg1B, regenerating family member 1 beta; EIEC, enteroinvasive *E. coli*.


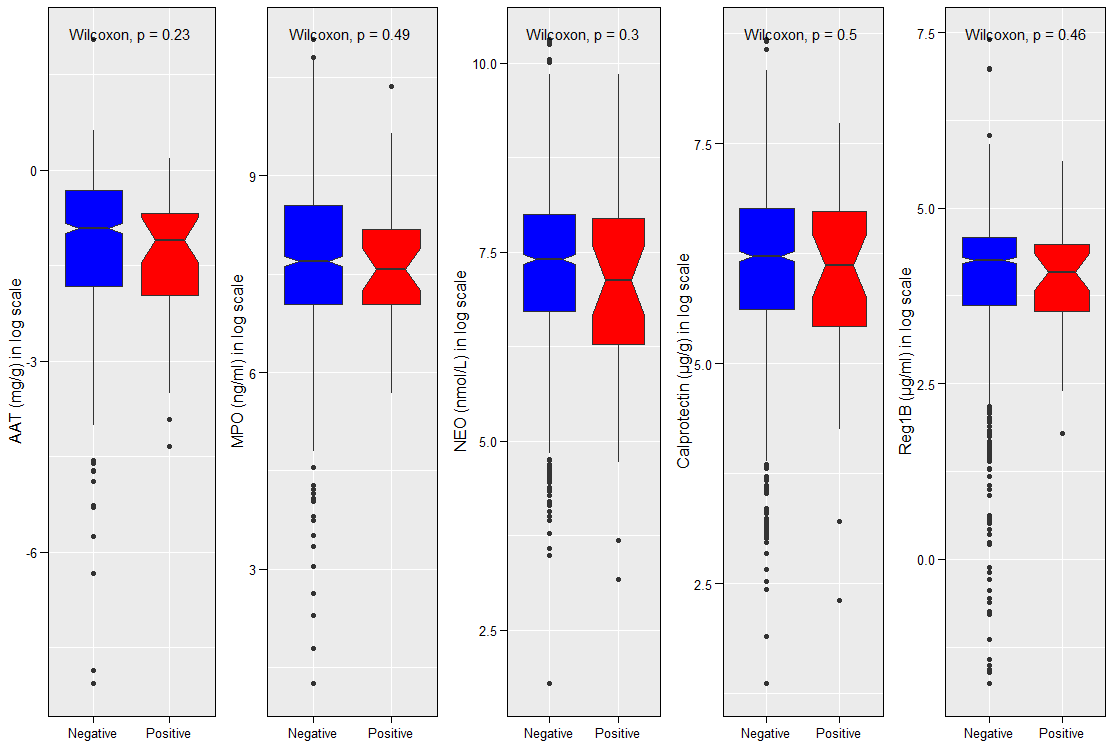


**Figure S5:** Distribution of fecal biomarkers in children (n = 1030) with and without STEC infection. A1AT, alpha-1 antitrypsin; MPO, myeloperoxidase; NEO, neopterin; Reg1B, regenerating family member 1 beta; STEC, Shiga toxin-producing *E. coli*.
