# Supplementary material for: Infection With Escherichia Coli Pathotypes Is Associated With Biomarkers of Gut Enteropathy and Nutritional Status Among Malnourished Children in Bangladesh
Source: Front Cell Infect Microbiol. 2022 Jul 6;12:901324. doi: 10.3389/fcimb.2022.901324 (PMC9299418; doi:10.3389/fcimb.2022.901324)
Supplement: Supplementary file 2 [file Table_1.docx]

**Table S1: Distribution of fecal biomarkers among children with and without *E. coli* pathotypes**

|  | **EAEC** | |
| --- | --- | --- |
|  | **No** | **Yes** |
|  | **(n=325)** | **(n=717)** |
| AAT, Median [Q1, Q3] (mg/g) | 0.38 [0.16, 0.71] | 0.39 [0.16, 0.72] |
| Calprotectin, Median [Q1, Q3] (μg/g) | 477 [230, 795] | 522 [289, 911] |
| MPO, Median [Q1, Q3] (ng/mL) | 1940 [1030, 4360] | 2340 [1170, 5460] |
| NEO, Median [Q1, Q3] (nmol/L) | 1650 [824, 2860] | 1640 [811, 2990] |
| REG1B, Median [Q1, Q3] μg/mL | 65.2 [27.2, 94.3] | 72.3 [41.1, 98.8] |
|  | **ETEC** | |
|  | **No** | **Yes** |
|  | **(n=583)** | **(n=459)** |
| AAT, Median [Q1, Q3] (mg/g) | 0.39 [0.16, 0.70] | 0.39 [0.16, 0.74] |
| Calprotectin, Median [Q1, Q3] (μg/g) | 467 [249, 819] | 555 [298, 920] |
| MPO, Median [Q1, Q3] (ng/mL) | 2200 [1030, 5510] | 2120 [1200, 4640] |
| NEO, Median [Q1, Q3] (nmol/L) | 1860 [858, 3190] | 1490 [755, 2680] |
| REG1B, Median [Q1, Q3] μg/mL | 70.6 [35.6, 97.4] | 69.6 [38.7, 96.6] |
|  | **STEC** | |
|  | **No** | **Yes** |
|  | **(n=1009)** | **(n=33)** |
| AAT, Median [Q1, Q3] (mg/g) | 0.40 [0.16, 0.72] | 0.33 [0.14, 0.51] |
| Calprotectin, Median [Q1, Q3] (μg/g) | 501 [275, 871] | 452 [226, 839] |
| MPO, Median [Q1, Q3] (ng/mL) | 2200 [1150, 5130] | 1950 [1140, 3600] |
| NEO, Median [Q1, Q3] (nmol/L) | 1650 [826, 2970] | 1240 [535, 2850] |
| REG1B, Median [Q1, Q3] μg/mL | 70.4 [37.4, 97.3] | 59.8 [34.2, 88.1] |
|  | **Shigella/EIEC** | |
|  | **No** | **Yes** |
|  | **(n=840)** | **(n=202)** |
| AAT, Median [Q1, Q3] (mg/g) | 0.40 [0.16, 0.73] | 0.38 [0.14, 0.68] |
| Calprotectin, Median [Q1, Q3] (μg/g) | 485 [260, 847] | 584 [338, 886] |
| MPO, Median [Q1, Q3] (ng/mL) | 2100 [1130, 4600] | 2490 [1170, 6400] |
| NEO, Median [Q1, Q3] (nmol/L) | 1750 [843, 3070] | 1250 [719, 2610] |
| REG1B, Median [Q1, Q3] μg/mL | 69.0 [36.9, 96.6] | 76.8 [39.2, 97.5] |
|  | **EPEC** | |
|  | **No** | **Yes** |
|  | **(n=460)** | **(n=582)** |
| AAT, Median [Q1, Q3] (mg/g) | 0.38 [0.15, 0.67] | 0.40 [0.16, 0.73] |
| Calprotectin, Median [Q1, Q3] (μg/g) | 470 [230, 840] | 529 [307, 880] |
| MPO, Median [Q1, Q3] (ng/mL) | 1950 [968, 5100] | 2410 [1260, 5050] |
| NEO, Median [Q1, Q3] (nmol/L) | 1660 [788, 2990] | 1640 [843, 2930] |
| REG1B, Median [Q1, Q3] μg/mL | 67.9 [37.5, 92.5] | 71.3 [37.1, 99.7] |

**Table S2: *E. coli* pathotypes and co-infection with other pathogens**

| **Pathogens** | **EAEC** | | | | **ETEC** | | | **Shigella/EIEC** | | | | **STEC** | | | **EPEC** | | |
| --- | --- | --- | --- | --- | --- | --- | --- | --- | --- | --- | --- | --- | --- | --- | --- | --- | --- |
|  | Negative | Positive | p-value | Negative | | Positive | p-value | Negative | Positive | p-value | Negative | | Positive | p-value | Negative | Positive | p-value |
| B.fragilis, n (%) | 47 (14.46) | 122 (17.02) | 0.300 | 97 (16.64) | | 72 (15.72) | 0.690 | 136 (16.19) | 33 (16.34) | 0.960 | 159 (15.76) | | 10 (30.30) | 0.026 | 67 (14.57) | 102 (17.53) | 0.198 |
| Sapovirus, n (%) | 50 (15.63) | 134 (18.85) | 0.211 | 108 (18.72) | | 76 (16.72) | 0.410 | 144 (17.35) | 40 (19.90) | 0.397 | 180 (18.02) | | 4 (12.50) | 0.422 | 75 (16.45) | 109 (18.96) | 0.296 |
| Adenovirus, n (%) | 164 (50.46) | 363 (50.63) | 0.960 | 285 (48.89) | | 241 (52.62) | 0.232 | 420 (50) | 107 (52.97) | 0.448 | 507 (50.25) | | 20 (60.61) | 0.242 | 232 (50.43) | 295 (50.69) | 0.935 |
| Campylobacter, n (%) | 164 (50.46) | 387 (53.97) | 0.293 | 282 (48.37) | | 269 (58.73) | 0.001 | 426 (50.71) | 125 (61.88) | 0.004 | 531 (52.63) | | 20 (60.61) | 0.366 | 232 (50.43) | 319 (54.81) | 0.160 |
| Cryptosporidium, n (%) | 45 (13.85) | 58 (8.09) | 0.004 | 62 (10.63) | | 41 (8.95) | 0.367 | 83 (9.88) | 20 (9.90) | 0.993 | 99 (9.81) | | 4 (12.12) | 0.662 | 44 (9.57) | 59 (10.14) | 7.590 |
| Norovirus, n (%) | 68 (21.25) | 168 (23.66) | 0.394 | 127 (22.05) | | 109 (24.01) | 0.457 | 190 (22.92) | 46 (22.89) | 0.992 | 228 (22.85) | | 8 (25) | 0.775 | 104 (22.81) | 132 (23) | 0.943 |
| Giardia, n (%) | 23 (7.10) | 46 (6.42) | 0.682 | 35 (6.00) | | 34 (7.44) | 0.356 | 50 (5.96) | 19 (9.41) | 0.077 | 64 (6.35) | | 5 (15.15) | 0.045 | 28 (6.09) | 41 (7.06) | 0.532 |
| EAEC, n (%) | - | - | - | 382 (65.52) | | 335 (73.14) | 0.008 | 571 (67.98) | 146 (72.28) | 0.236 | 692 (68.58) | | 25 (75.76) | 0.381 | 283 (61.52) | 434 (74.57) | <0.001 |
| ETEC, n (%) | 123 (37.96) | 335 (46.72) | 0.008 | - | | - | - | 349 (41.60) | 109 (53.96) | 0.001 | 439 (43.55) | | 19 (57.58) | 0.110 | 166 (36.09) | 292 (50.26) | <0.001 |
| Shigella/EIEC, n (%) | 56 (17.23) | 146 (20.36) | 0.236 | 93 (15.95) | | 109 (23.80) | 0.001 | - | - | - | 189 (18.73) | | 13 (39.39) | 0.003 | 84 (18.26) | 118 (20.27) | 0.414 |
| STEC, n (%) | 8 (2.46) | 25 (3.49) | 0.381 | 14 (2.40) | | 19 (4.15) | 0.110 | 20 (2.38) | 13 (6.44) | 0.003 | - | | - | - | 30 (6.52) | 3 (0.52) | <0.001 |
| EPEC, n (%) | 148 (45.54) | 434 (60.53) | <0.001 | 289 (49.57) | | 292 (63.76) | <0.001 | 464 (55.24) | 118 (58.42) | 0.414 | 579 (57.38) | | 3 (9.09) | <0.001 | - | - | - |

**Table S3: Association of *E. coli* pathotypes with the fecal biomarkers of EED using multivariable linear regression analysis ¶ (n=1028).**

| **Variables** | **AAT** | **MPO** | **NEO** | **Calprotectin** | **Reg1B** |
| --- | --- | --- | --- | --- | --- |
| Age in months | 0.02 [-0.03; 0.06] | -0.05 [-0.09; -0.01] ^*^ | -0.08 [-0.11; -0.05] ^*^ | -0.02 [-0.05; 0.01] | -0.05 [-0.08; -0.02] ^*^ |
| Sex (female) | 0.06 [-0.12; 0.24] | 0.05 [-0.11; 0.21] | -0.01 [-0.14; 0.13] | 0.05 [-0.07; 0.18] | -0.02 [-0.16; 0.12] |
| Water treatment | 0.00 [-0.19; 0.20] | 0.12 [-0.06; 0.30] | -0.07 [-0.22; 0.08] | 0.02 [-0.12; 0.16] | -0.00 [-0.16; 0.16] |
| Improved sanitation | -0.04 [-0.23; 0.15] | 0.08 [-0.08; 0.25] | 0.10 [-0.04; 0.24] | -0.00 [-0.14; 0.13] | -0.02 [-0.16; 0.13] |
| Monthly family income | 0.00 [-0.00; 0.00] | 0.00 [-0.00; 0.00] | 0.00 [-0.00; 0.00] | -0.00 [-0.00; 0.00] | -0.00 [-0.00; 0.00] |
| Mothers received education | -0.00 [-0.03; 0.03] | -0.02 [-0.05; 0.00] | -0.00 [-0.03; 0.02] | -0.02 [-0.04; -0.00] ^*^ | 0.02 [-0.01; 0.04] |
| Asset score | -0.00 [-0.07; 0.06] | -0.02 [-0.08; 0.03] | 0.02 [-0.03; 0.07] | 0.03 [-0.02; 0.07] | 0.00 [-0.05; 0.05] |
| Always wash hand with soap after child defecation | 0.11 [-0.13; 0.34] | -0.07 [-0.28; 0.14] | -0.17 [-0.34; 0.01] | 0.01 [-0.16; 0.17] | 0.14 [-0.05; 0.32] |
| Always wash hand with soap before preparing food | -0.10 [-0.39; 0.18] | -0.13 [-0.39; 0.12] | -0.10 [-0.32; 0.11] | -0.04 [-0.24; 0.16] | -0.03 [-0.26; 0.19] |
| Always wash hand with soap after using toilet | -0.13 [-0.38; 0.13] | 0.13 [-0.10; 0.36] | 0.07 [-0.12; 0.27] | -0.07 [-0.25; 0.11] | -0.05 [-0.25; 0.16] |
| Always use toilet paper | -0.12 [-0.36; 0.12] | -0.08 [-0.29; 0.14] | -0.04 [-0.22; 0.14] | 0.05 [-0.12; 0.22] | -0.07 [-0.26; 0.12] |
| Infection with EAEC | 0.07 [-0.12; 0.27] | 0.10 [-0.07; 0.28] | 0.02 [-0.12; 0.17] | 0.12 [-0.02; 0.26] | **0.28 [ 0.12; 0.43] ^*^** |
| Infection with ETEC | -0.04 [-0.22; 0.14] | -0.01 [-0.17; 0.15] | **-0.16 [-0.30; -0.02] ^*^** | **0.14 [ 0.01; 0.26] ^*^** | -0.06 [-0.20; 0.08] |
| Infection with Shigella/EIEC | 0.00 [-0.23; 0.23] | 0.15 [-0.05; 0.36] | -0.13 [-0.30; 0.04] | 0.13 [-0.03; 0.30] | 0.06 [-0.12; 0.24] |
| Infection with STEC | -0.14 [-0.65; 0.37] | -0.01 [-0.47; 0.44] | -0.19 [-0.58; 0.20] | -0.16 [-0.52; 0.21] | -0.03 [-0.44; 0.37] |
| Infection with EPEC | -0.08 [-0.26; 0.11] | 0.12 [-0.05; 0.28] | 0.06 [-0.08; 0.20] | 0.10 [-0.03; 0.23] | 0.02 [-0.13; 0.16] |
| AGP | 0.00 [-0.00; 0.00] | 0.00 [-0.00; 0.00] | -0.00 [-0.00; 0.00] | 0.00 [-0.00; 0.00] | -0.00 [-0.00; 0.00] |
| CRP | 0.00 [-0.01; 0.02] | 0.01 [ 0.00; 0.02] ^*^ | 0.01 [ 0.00; 0.02] ^*^ | 0.01 [-0.00; 0.01] | -0.00 [-0.01; 0.00] |
| Zinc | 0.56 [-0.10; 1.22] | 0.71 [ 0.12; 1.31] ^*^ | 0.39 [-0.11; 0.89] | 0.35 [-0.12; 0.82] | 0.31 [-0.21; 0.83] |
| Ferritin | 0.00 [-0.00; 0.00] | -0.00 [-0.00; 0.00] | 0.00 [-0.00; 0.00] | -0.00 [-0.00; 0.00] | 0.00 [-0.00; 0.00] |
| Adjusted in linear regression model for age, sex, WAMI Index (water/sanitation, assets, maternal education, and income); wash indices (hand wash with soap after helping child defecate, hand wash with soap before preparing food, hand wash with soap after using toilet, always use toilet paper); water treatment; individual *E. coli* pathotypes; inflammatory biomarkers (AGP, CRP); zinc; ferritin.  Dependent variable: AAT; MPO; NEO; calprotectin; Reg1B; Independent variables: Infection with E. coli pathotypes (EAEC, ETEC, Shigella/EIEC, STEC, EPEC).  ¶Each column represents an individual model. Biomarker values were log-transformed prior to analysis. The adjusted coefficient with 95% confidence interval (CI) has been reported. The asterisk (*) denotes the statistical significance with a p-value < 0.05.  Abbreviations used: AAT, alpha-1 antitrypsin; MPO, myeloperoxidase; NEO, neopterin; Reg1B, regenerating family member 1 beta; EAEC, enteroaggregative *E. coli*; ETEC, enterotoxigenic *E. coli*; EIEC, enteroinvasive *E. coli*; STEC, Shiga toxin-producing *E. coli*; EPEC, enteropathogenic *E. coli*; CRP, C-reactive protein; AGP, Alpha-1-acid glycoprotein. | | | | | |

**Table S4: Association of *E. coli* pathotypes (with sub-types) with the fecal biomarkers of EED using multivariable linear regression analysis ¶ (n=1028).**

| **Variables** | **AAT** | **MPO** | **NEO** | **Calprotectin** | **Reg1B** |  |
| --- | --- | --- | --- | --- | --- | --- |
| Age in months | 0.02 [-0.02; 0.06] | -0.05 [-0.09; -0.01] ^*^ | -0.08 [-0.11; -0.05] ^*^ | -0.02 [-0.05; 0.01] | -0.05 [-0.08; -0.01] ^*^ |  |
| Sex (female) | 0.06 [-0.11; 0.24] | 0.05 [-0.10; 0.21] | -0.01 [-0.14; 0.13] | 0.05 [-0.07; 0.18] | -0.02 [-0.16; 0.12] |  |
| Water treatment | -0.01 [-0.21; 0.19] | 0.11 [-0.07; 0.29] | -0.08 [-0.23; 0.07] | 0.02 [-0.12; 0.16] | -0.00 [-0.16; 0.15] |  |
| Improved sanitation | -0.04 [-0.23; 0.14] | 0.09 [-0.08; 0.26] | 0.11 [-0.03; 0.25] | -0.02 [-0.16; 0.11] | -0.02 [-0.17; 0.13] |  |
| Monthly family income | 0.00 [-0.00; 0.00] | 0.00 [-0.00; 0.00] | 0.00 [-0.00; 0.00] | -0.00 [-0.00; 0.00] | -0.00 [-0.00; 0.00] |  |
| Mothers received education | -0.00 [-0.03; 0.03] | -0.02 [-0.05; 0.00] | -0.00 [-0.03; 0.02] | -0.02 [-0.04; -0.00] ^*^ | 0.02 [-0.01; 0.04] |  |
| Asset score | 0.00 [-0.06; 0.06] | -0.02 [-0.08; 0.03] | 0.02 [-0.03; 0.07] | 0.03 [-0.02; 0.07] | 0.00 [-0.05; 0.05] |  |
| Always wash hand with soap after child defecation | 0.11 [-0.13; 0.34] | -0.07 [-0.28; 0.14] | -0.17 [-0.35; 0.01] | 0.00 [-0.16; 0.17] | 0.14 [-0.05; 0.33] |  |
| Always wash hand with soap before preparing food | -0.12 [-0.41; 0.16] | -0.14 [-0.40; 0.12] | -0.11 [-0.33; 0.11] | -0.03 [-0.23; 0.18] | -0.03 [-0.26; 0.20] |  |
| Always wash hand with soap after using toilet | -0.14 [-0.39; 0.12] | 0.13 [-0.10; 0.36] | 0.07 [-0.12; 0.27] | -0.07 [-0.25; 0.11] | -0.05 [-0.25; 0.15] |  |
| Always use toilet paper | -0.12 [-0.36; 0.12] | -0.08 [-0.30; 0.14] | -0.04 [-0.22; 0.15] | 0.05 [-0.12; 0.22] | -0.07 [-0.26; 0.12] |  |
| Infection with EAEC | 0.09 [-0.10; 0.28] | 0.11 [-0.07; 0.28] | 0.03 [-0.12; 0.17] | 0.12 [-0.01; 0.26] | **0.28 [ 0.13; 0.44] ^*^** |  |
| Infection with ST-ETEC | -0.03 [-0.24; 0.18] | 0.02 [-0.17; 0.21] | -0.13 [-0.29; 0.03] | 0.05 [-0.09; 0.20] | -0.08 [-0.24; 0.09] |  |
| Infection with LT-ETEC | -0.16 [-0.41; 0.10] | -0.09 [-0.32; 0.14] | **-0.21 [-0.40; -0.02] ^*^** | **0.26 [ 0.07; 0.44] ^*^** | -0.06 [-0.27; 0.14] |  |
| Infection with Shigella/EIEC | -0.01 [-0.24; 0.22] | 0.14 [-0.06; 0.35] | -0.13 [-0.31; 0.04] | 0.15 [-0.01; 0.31] | 0.06 [-0.12; 0.24] |  |
| Infection with STEC | -0.17 [-0.68; 0.34] | -0.03 [-0.48; 0.43] | -0.19 [-0.58; 0.19] | -0.15 [-0.51; 0.21] | -0.04 [-0.45; 0.36] |  |
| Infection with aEPEC | -0.18 [-0.38; 0.02] | 0.09 [-0.09; 0.27] | 0.06 [-0.09; 0.21] | 0.08 [-0.06; 0.23] | -0.03 [-0.19; 0.13] |  |
| Infection with tEPEC | 0.15 [-0.11; 0.40] | 0.18 [-0.05; 0.41] | 0.06 [-0.14; 0.25] | 0.17 [-0.01; 0.36] | 0.13 [-0.07; 0.34] |  |
| AGP | 0.00 [-0.00; 0.00] | 0.00 [-0.00; 0.00] | -0.00 [-0.00; 0.00] | 0.00 [-0.00; 0.00] | -0.00 [-0.00; 0.00] |  |
| CRP | 0.00 [-0.01; 0.02] | 0.01 [ 0.00; 0.02] ^*^ | 0.01 [ 0.00; 0.02] ^*^ | 0.01 [-0.00; 0.01] | -0.00 [-0.01; 0.00] |  |
| Zinc | 0.60 [-0.06; 1.26] | 0.73 [ 0.13; 1.32] ^*^ | 0.39 [-0.11; 0.89] | 0.36 [-0.11; 0.83] | 0.33 [-0.20; 0.85] |  |
| ferritin | 0.00 [-0.00; 0.00] | -0.00 [-0.00; 0.00] | 0.00 [-0.00; 0.00] | -0.00 [-0.00; 0.00] | 0.00 [-0.00; 0.00] |  |
| Adjusted in linear regression model for age, sex, WAMI Index (water/sanitation, assets, maternal education, and income); wash indices (hand wash with soap after helping child defecate, hand wash with soap before preparing food, hand wash with soap after using toilet, always use toilet paper); water treatment; individual *E. coli* pathotypes; inflammatory biomarkers (AGP, CRP); zinc; ferritin.  Dependent variable: AAT; MPO; NEO; calprotectin; Reg1B; Independent variables: Infection with E. coli pathotypes (EAEC, ETEC, Shigella/EIEC, STEC, EPEC).  ¶Each column represents an individual model. Biomarker values were log-transformed prior to analysis. The adjusted coefficient with 95% confidence interval (CI) has been reported. The asterisk (*) denotes the statistical significance with a p-value < 0.05.  Abbreviations used: AAT, alpha-1 antitrypsin; MPO, myeloperoxidase; NEO, neopterin; Reg1B, regenerating family member 1 beta; EAEC, enteroaggregative *E. coli*; ETEC, enterotoxigenic *E. coli*; EIEC, enteroinvasive *E. coli*; STEC, Shiga toxin-producing *E. coli*; EPEC, enteropathogenic *E. coli*; CRP, C-reactive protein; AGP, Alpha-1-acid glycoprotein. | | | | | | |

**Table S5: Association of *E. coli* pathotypes with the indicators of nutritional status in children ¶ (n=1028)**

| **Variables** | **LAZ** | **WAZ** | **WLZ** |
| --- | --- | --- | --- |
| AAT | 0.06 [ 0.03; 0.10] ^*^ | 0.02 [-0.01; 0.06] | -0.02 [-0.06; 0.02] |
| MPO | -0.11 [-0.15; -0.08] ^*^ | -0.07 [-0.11; -0.02] ^*^ | -0.00 [-0.05; 0.04] |
| NEO | 0.08 [ 0.04; 0.12] ^*^ | 0.03 [-0.02; 0.07] | -0.02 [-0.07; 0.03] |
| Calprotectin | 0.08 [ 0.04; 0.13] ^*^ | 0.05 [-0.00; 0.10] | 0.01 [-0.05; 0.06] |
| Reg1B | 0.04 [-0.01; 0.08] | 0.03 [-0.02; 0.07] | 0.01 [-0.03; 0.06] |
| Age in months | -0.02 [-0.04; 0.01] | -0.01 [-0.04; 0.01] | -0.03 [-0.05; 0.00] |
| Sex (female) | 0.20 [ 0.11; 0.29] ^*^ | 0.12 [ 0.02; 0.22] ^*^ | 0.06 [-0.05; 0.17] |
| Water treatment | 0.10 [-0.00; 0.20] | 0.14 [ 0.02; 0.25] ^*^ | 0.11 [-0.01; 0.23] |
| Improved sanitation | 0.02 [-0.07; 0.12] | 0.11 [ 0.00; 0.22] ^*^ | 0.13 [ 0.01; 0.24] ^*^ |
| Monthly family income | 0.00 [-0.00; 0.00] | 0.00 [-0.00; 0.00] | 0.00 [-0.00; 0.00] |
| Mothers received education | 0.02 [ 0.01; 0.04] ^*^ | 0.03 [ 0.01; 0.05] ^*^ | 0.03 [ 0.01; 0.04] ^*^ |
| Asset score | 0.03 [-0.01; 0.06] | 0.06 [ 0.02; 0.09] ^*^ | 0.06 [ 0.02; 0.10] ^*^ |
| Always wash hand with soap after child defecation | -0.04 [-0.16; 0.09] | 0.04 [-0.09; 0.17] | 0.08 [-0.07; 0.22] |
| Always wash hand with soap before preparing food | -0.01 [-0.16; 0.13] | -0.07 [-0.23; 0.10] | -0.07 [-0.25; 0.10] |
| Always wash hand with soap after using toilet | 0.06 [-0.07; 0.19] | -0.03 [-0.18; 0.11] | -0.10 [-0.26; 0.05] |
| Always use toilet paper | 0.06 [-0.07; 0.18] | 0.01 [-0.13; 0.14] | -0.04 [-0.18; 0.11] |
| Infection with EAEC | 0.03 [-0.07; 0.13] | 0.06 [-0.05; 0.17] | 0.05 [-0.06; 0.17] |
| Infection with ETEC | 0.09 [-0.00; 0.18] | 0.05 [-0.05; 0.16] | 0.01 [-0.10; 0.12] |
| Infection with Shigella/EIEC | 0.02 [-0.10; 0.13] | -0.03 [-0.16; 0.10] | -0.06 [-0.20; 0.07] |
| Infection with STEC | -0.18 [-0.44; 0.08] | -0.07 [-0.36; 0.22] | 0.04 [-0.27; 0.35] |
| Infection with EPEC | **-0.12 [-0.22; -0.03] ^*^** | **-0.11 [-0.22; -0.01] ^*^** | -0.06 [-0.17; 0.05] |
| Zinc | 0.04 [-0.30; 0.37] | 0.08 [-0.29; 0.46] | 0.06 [-0.34; 0.47] |
| Ferritin | 0.00 [-0.00; 0.00] | -0.00 [-0.00; 0.00] | -0.00 [-0.00; 0.00] |
| Adjusted in linear regression model for age, sex, WAMI Index (water/sanitation, assets, maternal education, and income); wash indices (hand wash with soap after helping child defecate, hand wash with soap before preparing food, hand wash with soap after using toilet, always use toilet paper); individual *E. coli* pathotypes; water treatment; EED biomarkers (AAT, MPO, NEO, calprotectin, Reg1B); maternal height; zinc; ferritin.  Dependent variable: LAZ; WAZ; WLZ; Independent variables: Infection with E. coli pathotypes.  ^¶^Each column represents an individual model. Biomarker values were log-transformed prior to analysis. The adjusted coefficient with 95% confidence interval (CI) has been reported. The asterisk (*) denotes the statistical significance with a p-value < 0.05.  Abbreviations used: LAZ, length-for-age; WAZ, weight-for-age; WLZ, weight-for-length | | | |

**Table S6: Association of *E. coli* pathotypes (with sub-types) with the indicators of nutritional status in children ¶ (n=1028)**

|  | **LAZ** | **WAZ** | **WLZ** |
| --- | --- | --- | --- |
| AAT | 0.06 [ 0.03; 0.10]^*^ | 0.02 [-0.01; 0.06] | -0.02 [-0.06; 0.02] |
| MPO | -0.12 [-0.15; -0.08]^*^ | -0.07 [-0.11; -0.02]^*^ | -0.00 [-0.05; 0.04] |
| NEO | 0.08 [ 0.04; 0.12]^*^ | 0.03 [-0.02; 0.07] | -0.02 [-0.07; 0.03] |
| Calprotectin | 0.08 [ 0.04; 0.13]^*^ | 0.05 [-0.00; 0.10] | 0.00 [-0.05; 0.06] |
| Reg1B | 0.04 [-0.00; 0.08] | 0.03 [-0.01; 0.07] | 0.02 [-0.03; 0.06] |
| Age in months | -0.02 [-0.04; 0.01] | -0.01 [-0.04; 0.01] | -0.03 [-0.05; 0.00] |
| Sex (female) | 0.20 [ 0.11; 0.29]^*^ | 0.12 [ 0.02; 0.22]^*^ | 0.06 [-0.05; 0.17] |
| Water treatment | 0.09 [-0.01; 0.20] | 0.14 [ 0.02; 0.25]^*^ | 0.12 [-0.00; 0.24] |
| Improved sanitation | 0.03 [-0.07; 0.12] | 0.11 [ 0.00; 0.22]^*^ | 0.12 [ 0.01; 0.24]^*^ |
| Monthly family income | 0.00 [-0.00; 0.00] | 0.00 [-0.00; 0.00] | 0.00 [-0.00; 0.00] |
| Mothers received education | 0.02 [ 0.01; 0.04]^*^ | 0.03 [ 0.01; 0.05]^*^ | 0.03 [ 0.01; 0.04]^*^ |
| Asset score | 0.03 [-0.00; 0.06] | 0.06 [ 0.02; 0.09]^*^ | 0.06 [ 0.02; 0.09]^*^ |
| Always wash hand with soap after child defecation | -0.04 [-0.16; 0.09] | 0.04 [-0.09; 0.17] | 0.08 [-0.07; 0.22] |
| Always wash hand with soap before preparing food | -0.02 [-0.17; 0.13] | -0.07 [-0.23; 0.10] | -0.07 [-0.25; 0.11] |
| Always wash hand with soap after using toilet | 0.06 [-0.07; 0.19] | -0.03 [-0.18; 0.11] | -0.10 [-0.25; 0.06] |
| Always use toilet paper | 0.06 [-0.07; 0.18] | 0.01 [-0.13; 0.14] | -0.03 [-0.18; 0.11] |
| Infection with EAEC | 0.03 [-0.07; 0.13] | 0.06 [-0.05; 0.17] | 0.05 [-0.07; 0.17] |
| Infection with ST-ETEC | **0.12 [ 0.01; 0.23]^*^** | 0.05 [-0.07; 0.17] | -0.01 [-0.14; 0.11] |
| Infection with LT-ETEC | 0.04 [-0.09; 0.17] | 0.07 [-0.08; 0.21] | 0.06 [-0.09; 0.22] |
| Infection with Shigella/EIEC | 0.01 [-0.11; 0.13] | -0.02 [-0.15; 0.11] | -0.06 [-0.20; 0.08] |
| Infection with STEC | -0.18 [-0.44; 0.08] | -0.06 [-0.35; 0.22] | 0.04 [-0.27; 0.35] |
| Infection with aEPEC | **-0.12 [-0.23; -0.02]^*^** | -0.10 [-0.21; 0.02] | -0.04 [-0.17; 0.08] |
| Infection with tEPEC | -0.13 [-0.26; 0.00] | -0.14 [-0.29; 0.00] | -0.10 [-0.25; 0.06] |
| Zinc | 0.04 [-0.30; 0.38] | 0.08 [-0.30; 0.46] | 0.06 [-0.35; 0.46] |
| ferritin | 0.00 [-0.00; 0.00] | -0.00 [-0.00; 0.00] | -0.00 [-0.00; 0.00] |
| Adjusted in linear regression model for age, sex, WAMI Index (water/sanitation, assets, maternal education, and income); wash indices (hand wash with soap after helping child defecate, hand wash with soap before preparing food, hand wash with soap after using toilet, always use toilet paper); individual *E. coli* pathotypes; water treatment; EED biomarkers (AAT, MPO, NEO, calprotectin, Reg1B); maternal height; zinc; ferritin.  Dependent variable: LAZ; WAZ; WLZ; Independent variables: Infection with E. coli pathotypes.  ^¶^Each column represents an individual model. Biomarker values were log-transformed prior to analysis. The adjusted coefficient with 95% confidence interval (CI) has been reported. The asterisk (*) denotes the statistical significance with a p-value < 0.05.  Abbreviations used: LAZ, length-for-age; WAZ, weight-for-age; WLZ, weight-for-length | | | |
